# Real-Time Recognition Algorithm of Small Target for UAV Infrared Detection

**DOI:** 10.3390/s24103075

**Published:** 2024-05-12

**Authors:** Qianqian Zhang, Li Zhou, Junshe An

**Affiliations:** 1 National Space Science Center, Chinese Academy of Sciences, Beijing 101499, China; zhangqianqian21@mails.ucas.ac.cn (Q.Z.); anjunshe@nssc.ac.cn (J.A.); 2 School of Computer Science and Technology, University of Chinese Academy of Sciences, Beijing 100049, China; 3 School of Astronomy and Space Science, University of Chinese Academy of Sciences, Beijing 100049, China

**Keywords:** UAV, infrared, small target, picodet, detection, real-time

## Abstract

Unmanned Aerial Vehicle (UAV) infrared detection has problems such as weak and small targets, complex backgrounds, and poor real-time detection performance. It is difficult for general target detection algorithms to achieve the requirements of a high detection rate, low missed detection rate, and high real-time performance. In order to solve these problems, this paper proposes an improved small target detection method based on Picodet. First, to address the problem of poor real-time performance, an improved lightweight LCNet network was introduced as the backbone network for feature extraction. Secondly, in order to solve the problems of high false detection rate and missed detection rate due to weak targets, the Squeeze-and-Excitation module was added and the feature pyramid structure was improved. Experimental results obtained on the HIT-UAV public dataset show that the improved detection model’s real-time frame rate increased by 31 fps and the average accuracy (MAP) increased by 7%, which proves the effectiveness of this method for UAV infrared small target detection.

## 1. Introduction

In recent years, Unmanned Aerial Vehicle (UAV) detection technology has become an important reconnaissance and detection method in the civilian and military fields due to its good flexibility, concealment, and efficiency. With the continuous development of deep learning, many good results have been achieved in dim and small target detection in UAV detection scenarios. For example, T. Zhao et al. [1] proposed an infrared vehicle detection algorithm for UAV, which achieves the purpose of car navigation by identifying the locations of multiple vehicles in UAV images. B. M et al. [2] proposed a UAV urban object detection system for medical use, which achieves the purpose of smart medical care by identifying ambulances, hospitals, and other medical-related objects in UAV images. X. Xie et al. [3] proposed a forest fire detection method based on dual-light visual images of UAV, which completes forest fire flame detection by processing visible light images and infrared images acquired by UAV. M. H. Hamzenejadi et al. [4] proposed a civilian UAV vehicle detection method based on improved YOLOv5. By improving the YOLOv5 model structure, adding an attention mechanism, and using adaptive boundary box regression loss function, high-precision real-time and fast detection was realized. R. V. Sri Hari et al. [5] proposed a UAV detection model for detecting small stones, nuts, metal sheets, and millimeter-sized objects on airport runways by using improved YOLOv5 to identify small targets to maintain runway safety. H. Zhao et al. [6] proposed a YOLOv7-sea network for maritime search and rescue missions, which uses drones to detect people, ships, and other small objects in open waters. Gansheng Wang [7] proposed an improved Yolov5n lightweight aerial photography military target detection model to identify ground military targets through UAV aerial photography. S. Ji et al. [8] proposed a highly automated UAV military equipment reconnaissance system for complex environments, which uses an improved YOLOv5 network to identify small targets such as armor, helicopters, and personnel on the battlefield.

T.-Y. Lin et al. [9] mentioned that small targets are defined as targets with a resolution of less than 32 × 32 pixels, medium targets are larger than 32 × 32 pixels and smaller than 96 × 96 pixels, and targets larger than 96 × 96 pixels are large targets.

At present, small target detection algorithms can be divided into neural network methods [10], space-time tensor methods [11,12,13], and low-rank sparse decomposition [14]. In terms of computational complexity, space-time tensor methods and low-rank sparse decomposition algorithms involve relatively complex mathematical operations and optimization processes. The algorithm complexity is high and it is difficult to meet real-time requirements. Especially when dealing with large-scale infrared image data, these problems may be more prominent. In terms of parameter sensitivity, they are often affected by parameter settings, and parameter selection usually requires experience or experimentation to determine. Inappropriate parameter settings may lead to inaccurate or unstable detection results. In terms of generalization ability, they have difficulty adapting to different scenarios and conditions. Such algorithms are usually based on specific assumptions, and these methods may need to be readjusted or designed when the target morphology, background, or noise changes. In contrast, deep learning algorithms can automatically learn feature information through network training, can reduce the amount of calculation by adjusting the network structure, and have the advantages of strong feature extraction and generalization capabilities, as well as high real-time performance.

**Figure 1:**
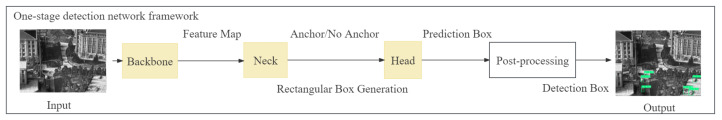
One -stage detection network framework.

**Figure 2:**
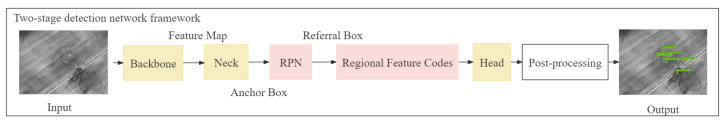
Two-stage detection network framework.

Currently, deep learning algorithms are divided into two categories: one-stage algorithms (see Figure 1) and two-stage algorithms (see Figure 2). Two-stage algorithms based on candidate regions, e.g., J. Ma et al. [15], used R-CNN networks to recognize field herbivores in UAV images with 97% mAp. Although R-CNN can achieve better results in terms of accuracy, the process of generating candidate regions by R-CNN is more time-consuming compared to SSD and YOLO, and the detection speed of R-CNN may become a bottleneck for its application in real-time applications. One-stage algorithms include SSD [16] and YOLO [17], etc. This type of algorithm adopts a more efficient region generation method to achieve faster detection speed, and it can improve the detection ability of small targets through multi-scale feature fusion and other methods.

However, due to the interference of point-like high-brightness noise and similar target interference objects on infrared images collected by UAV, especially in scene conditions with complex background radiation and clutter, it poses a major challenge to the task of long-distance identification of small targets. Moreover, due to the long imaging distance, the target usually appears as a dense small target, the target pixel proportion is small, the infrared image radiation intensity is weak, the signal-to-noise ratio is low, and the shape and texture information is very limited, which increases the false positive rate in the detection process. The low intensity characteristics of infrared images are mainly due to the properties of infrared radiation itself. The intensity of an infrared image depends on the temperature difference between the target and the background. If the temperature difference is small, the infrared radiation intensity of the target is low and difficult to distinguish from the background. The low signal-to-noise ratio of infrared images is caused by many factors. The infrared imaging process will be disturbed by various noises, such as background noise, circuit noise, and so on. In the case of low-intensity signals, the effect of electronic noise will be more obvious. This noise is superimposed on the infrared signal, reducing the signal’s signal-to-noise ratio. In addition, the currently commonly used YOLO detection network has problems such as a large number of model parameters, long detection time, and high deployment cost on the mobile terminal. For long-distance small-target detection tasks, it is difficult for general image processing technology to achieve the goals of high detection rate, low false alarm rate, and high real-time performance. Therefore, it is of great significance and application value to study how to accurately handle UAV infrared detection technology in various complex scenes.

Aiming at the problems of low detection accuracy caused by complex background interference, weak targets, and multi-scale in the infrared detection of small targets by UAV, an improved Picodet UAV infrared small target detection method is proposed.

Use cosine decay and linear preheating learning rate strategies to learn image features by gradually increasing the learning rate. Use the momentum optimizer to overcome noise and interference factors in the image to improve model performance. Based on the lightweight Picodet detection network, the LCNet network is used as the backbone network to fully extract the local and global features of the target while reducing the number of model parameters. The Squeeze-and-Excitation module and the improved feature pyramid structure are introduced to integrate shallow fine-grained features and high-level semantic features and expand the receptive field by fusing features of different scales, thereby improving the infrared small target detection performance.

The main work of this paper is as follows:

1. Aiming at the problem of real-time detection, an improved lightweight LCNet network is proposed as the feature extraction backbone network, which uses depth-separable convolution to fully extract small target features while reducing the amount of computation. This enables models to run more efficiently on platforms with limited memory and computing capabilities.
2. To solve the problem of low resolution and few features of small targets resulting in a high false detection rate, as well as the problem of large target scale span and coexistence of multiple scales leading to a high miss detection rate, the Squeeze-and-Excitation module was added and the feature pyramid structure was improved. The fusion of features at different scales expands the receptive field to enhance the accuracy of the network in identifying small targets.
3. Based on the image feature information characteristics of the HIT-UAV dataset, use cosine decay and linear preheating learning rate strategies to gradually increase the learning rate to better learn image features and improve detection accuracy and stability. The momentum optimizer helps the model better overcome noise and interference factors in images and improve detection performance.

## 2. Related Work

The target objects in the infrared detection data collected by UAV have the characteristics of small size, weak target intensity, and large number, which brings greater challenges to UAV infrared small target detection than ordinary target detection. Specifically, infrared image small targets have the following limitations in terms of shape and structure information: (1) Lack of features: Due to the small size of infrared small targets, they often occupy only a few pixels in the image, which leads to the lack of sufficient texture, structure, and other features. This makes it difficult for traditional target detection methods to extract effective feature information, which increases the difficulty of detection and recognition. (2) Low contrast: the infrared image itself has the characteristics of low signal-to-noise ratio and low contrast. When the temperature difference between the target and the background is small, the small target may fuse with the background, which makes the edge and shape information of the small target blurred and difficult to be clearly identified and distinguished. (3) Lack of color information: Infrared images are usually single-band and lack color information, which makes it impossible to rely on color features for recognition. (4) Susceptible to interference: the electronic noise of the infrared sensor and other noise sources may mask the weak signal of a small target, resulting in difficulty for detection algorithms to identify the real target. (5) Target occlusion: In complex scenes, small targets may be partially or completely occluded by other objects, resulting in incomplete target identification by detection algorithms. (6) Limitations of image processing algorithms: the image processing and object detection algorithms used may be unsuitable for processing the special characteristics of infrared images, such as the algorithm may not take into account the noise pattern or contrast characteristics of infrared images. To overcome these challenges, researchers have proposed many detection methods for small targets on UAV. These methods aim to improve the performance of network target detection and reduce the false alarm rate of small targets. These techniques include data augmentation, improved backbone networks, improved neck connection layers, improved anchor boxes, and improved loss functions.

**Figure 3:**
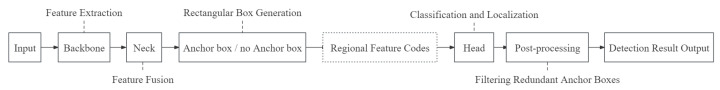
General target detection flow block diagram.

Usually, the process of target detection is as follows (see Figure 3): First, preprocessing operations such as data enhancement are performed on the dataset images. Secondly, it is sent to the backbone network to extract different scale features of the target. Enter the neck connection layer to fuse different scale features to generate multi-scale features. A rectangular frame of the predicted target is generated based on the set anchor frame size. Afterward, target detection is completed through regional feature encoding, classification and positioning, and loss functions.

Since the small target to be tested usually only occupies a small area in the images collected by the drone and has relatively little feature information, it is necessary to generate more training samples through data enhancement methods to help the model learn richer feature information. Commonly used data augmentation techniques include random cropping, flipping, scaling, and color transformation. Changsong Zhang et al. [18] expanded the training set images through data enhancement methods such as mosaic, translation, scaling, rotation, and color conversion to improve the generalization ability of the detection model for small target recognition. Gansheng Wang [7] reorganized images through random scaling, random cropping, and random splicing, which enhanced the richness of the military target dataset and improved the ability to identify small targets. Data enhancement can enable the model to adapt to different scenarios and conditions, and can better help the model generalize to unseen data, thereby improving the recognition ability of small targets.

The function of the backbone is to extract features of different scales of the target. Common lightweight backbone networks include: Mobilenet [19], ShuffleNet [20], GhostNet [21], and LCNet [22], etc. A. G. Howard et al. [19] of Google proposed the MobileNet network structure in 2017, balancing the size and performance of the model through depth-separable convolution and adjusting the two hyperparameters of width multiplier and resolution multiplier to enable efficient applications on mobile and embedded devices. In 2017, Facebook Company X. Zhang et al. [20] proposed the ShuffleNet network structure, which enhanced the information interaction between different channels by introducing the operation of channel shuffle. Meanwhile, group convolution and point-by-point convolution were adopted to reduce the computational complexity. K. Han et al. [21] of Huawei Noah’s Ark Laboratory proposed the GhostNet network structure in 2020, and introduced the Ghost module to generate feature maps to extract features at a small computational cost. C. Cui et al. [22] proposed a lightweight and high-performance network PP-LCNet in 2021, which achieves performance improvement with almost constant inference time by replacing the ReLU activation function with H-Swish. The balance of precision and speed is achieved by adding SE modules at appropriate positions at the end of the network. The model inference speed is improved by using only one size convolution kernel in a single layer. LCNet networks have lower latency and higher accuracy than ShuffleNetV2, MobileNetV2, MobileNetV3, and GhostNet. J. Chen et al. [23] adopted a lightweight network PP-LC network as the backbone network based on YOLOv4, and used depth separable convolution to reduce model parameters. Compared with the original YOLOv4, the accuracy was increased by 0.52%, the model size was reduced by about 83%, and the detection speed was increased by 88%.

The function of the neck connection layer is to fuse the different scale features extracted by the backbone to generate multi-scale features. Common feature fusion methods in the field of target detection mainly include cascade feature pyramid, feature pyramid pooling, feature pyramid fusion, multi-scale prediction, cross-layer feature fusion, and self-attention mechanism. S. Liu et al. [24] proposed a PANet network structure. By adding low-level features to the P2-P5 layers of FPN, the lowest-level features only need to pass through a few layers to flow to N2-N5, which is improved. The problem of long path for high and low layer feature fusion occurs in the FPN network. K. He et al. [25] first proposed the concept of spatial pyramid pooling (SPP), which allows the network to generate a fixed-size output when inputting pictures of any size, reducing the loss and deformation of picture features, and improving recognition accuracy. T.-Y. Lin et al. [26] proposed a feature pyramid fusion structure. Top-level features are fused with low-level features through upsampling. Each layer can be independently predicted, which significantly improves the performance of target detection. W. Liu et al. [27] used feature maps of different sizes for multi-scale prediction and detected targets at different scales, solving the problem of missed detection or false detection caused by the size of the target exceeding the detection range at a single scale. F. Yu et al. [28] proposed that the DLA structure fuses features of different layers through cross-layer connection and aggregation operations to improve the performance of target detection. W. Yi et al. [29] used YOLOv7 as the basic network to improve detection accuracy while reducing model complexity by integrating the SENet attention mechanism, enhancing the FPN network topology, and introducing the EIoU loss function.

The learning strategy is used to adjust the step size of parameters when training the network, which determines the degree of parameter adjustment at each gradient update and directly affects model performance. Commonly used learning rate adjustment strategies include piecewise constant decay, exponential decay, natural exponential decay, interval decay, multi-interval decay, inverse time decay, cosine decay, polynomial decay, Lambda decay, Noam decay, loss adaptive decay, etc. Qi Jiandong et al. [30] tested the impact of fixed learning strategy, segmented learning strategy, and cosine decay learning strategy on the accuracy of the BS-ResNeXt-50 network. After using the cosine annealing learning strategy, the accuracy of BS-ResNeXt50 increased to 81.54%, 3.5% higher than the fixed learning rate; the segmented learning rate accuracy reached 79.3%, which was 2.24% different from the cosine annealing learning rate. In addition, Linear Warm Up is a learning rate optimization strategy. The main idea is to gradually increase the learning rate before adjusting the learning rate normally. When the number of training steps is less than the number of warmup steps, the learning rate Lr is updated according to Formula (1). When the number of training steps is greater than or equal to the number of warmup steps, the learning rate Lr is updated according to Formula (2).

(1) $$lr=start\_lr+\left(end\_lr-start\_lr\right)*\frac{epoch}{warmup\_steps}$$

(2) lr=learning_rate

where lr is the learning rate after warm-up, start_lr is the initial value of the learning rate, end_lr is the final learning rate, and epoch is the number of training rounds. Tian T. et al. [31] used the learning strategy of first linear preheating and then cosine decays to conduct neural network model learning and training to detect small targets in granular sludge, which improved the problem of the difficult identification of small targets.

## 3. Methods and Datasets

This section is divided into three parts, introducing the overall structure and principles of the model in this article, the high-altitude infrared dataset HIT-UAV [32], and the evaluation indicators used to verify the method in this article.

### 3.1. Overall Model Structure

This section will introduce the overall structure and particular design of the proposed improved method.

**Figure 4:**
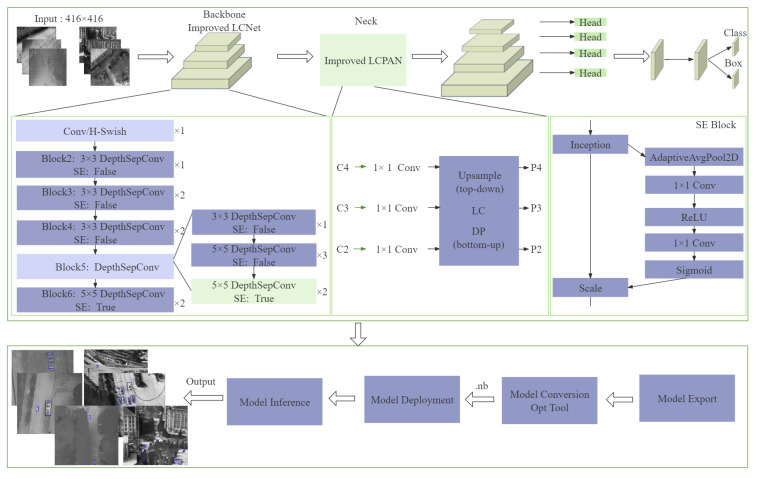
The overall block diagram of the specific technical implementation of the proposed method.

This paper proposes a real-time recognition network model of small targets for UAV infrared detection, as shown in Figure 4. The backbone network of this model adopts the LCNet network structure. Compared with similar lightweight algorithms, it can respond more quickly and process video streams or captured images in real-time. Compared with Mobilenet and Shufflenet networks, LCNet has fewer parameters. The neck network uses LCPAN. Compared with the commonly used CSPPAN network structure, LCPAN effectively improves network accuracy with a larger receptive field and fewer parameters. The PAN structure can obtain feature maps at multiple levels. At the same time, the LC structure can perform feature splicing and fusion between adjacent feature maps to obtain semantic and location information. The input of the LCPAN network is the multi-scale feature map generated by the backbone network, and then the LC module, DP module, and upsampling operation are used to perform top-down and bottom-up feature fusion, and finally the feature map is output. The LC module uses depthwise separable convolution as its basic building block. Finally, the obtained multi-scale feature map is used to obtain the prediction result through the picoheadV2 detection head.

#### 3.1.1. Improved Network Based on LCNet

Due to the relatively limited processing power and storage space of UAV, high-precision target detection models such as YOLOv8 are unsuitable for effective operation on UAV due to their high number of parameters and calculations. T. Aibibu et al. [33] gives that the mAP of YOLOv8 on the HIT-UAV dataset is 75.5%, while according to the data provided by the official YOLO, the number of references of [34] YOLOv8 is 68.2 M. Cui.C et al. [22] gives the LCNet parameter number without improvement as only 3.0 M. The number of parameters of LCNet network is much smaller than that of YOLOv8. Facing the airborne terminal deployment requirements for real-time processing and rapid identification, lightweight networks are the key to achieving efficient real-time identification. The LCNet network has lower latency and higher accuracy than ShuffleNetV2, MobileNetV2, MobileNetV3, and GhostNet.

The backbone network LCNet is a deep neural network composed of multiple convolution blocks, with a total of six blocks. Each block uses depth-separable convolution and optional SE modules to extract features, and outputs multi-scale feature maps at different levels through the forward propagation function.

The first block includes a convolutional layer and a batch normalization layer, as well as an activation function (hard_swish), blocks2 to blocks6, wherein each block has a different number of output channels and levels. These levels are different levels of the feature pyramid. Each layer corresponds to different feature sizes and resolutions and is used to capture information at different scales. Set the feature_maps parameter according to the characteristics of the dataset to specify the feature map level output by the network to output features of a specific level.

The depth-separable convolution layer includes depth convolution, optional SE module, and point-wise convolution. Using this type of convolution instead of traditional convolution can effectively reduce the number of parameters and the amount of calculation. The SE module consists of a global average pooling layer and two convolutional layers. The SE module reweights the channels so that the network can adaptively adjust the importance of different channels. This weighting mechanism helps the network focus on more important features and suppress unimportant features, thus enhancing the ability of feature representation. At the same time, the SE module promotes the generalization ability of the model to new data by learning the dependency relationship between channels, has good robustness in dealing with noise interference, and also ensures that the model can maintain high performance when dealing with unseen data. In addition, the SE module enhances the model’s ability to process targets at different scales by strengthening the global information of features. The feature pyramid structure helps to extract and use useful features at different levels, thus improving the model’s ability to recognize small and large targets. Finally, although the SE module significantly improves the performance of the network, the parameters and calculations it introduces are relatively low, which effectively improves the performance without significantly increasing the complexity of the model.

The table illustrates the network structure configuration before (see Table 1) and after the improvement (see Table 2). It includes different levels of convolution blocks (‘blocks1’ to ‘blocks6’). The configuration of each level includes the convolution kernel size (k), step size (s), and whether to use the SE module.

In the LCNet network, as the block number increases (from blocks1 to blocks6), the size of the feature map gradually decreases and the number of channels gradually increases. Therefore, to improve small target detection capabilities, the best place to add SE modules is usually at the deeper level of the network, which allows the network to better focus on small-scale features. The original model only adds the SE module in blocks6. Although this is the deepest level in the network, the feature map size is the smallest and can theoretically capture the most detailed features. However, due to the reduction in feature map size, more noise may be introduced, and the features may be too abstract, thus affecting the detection performance of small targets. This article also adds SE modules in blocks5. Adding SE modules at these levels can more fully capture the detailed features of small targets, enhance the network’s learning ability of small-scale features, and improve the detection effect of small targets.

The feature pyramid structure enables the network to capture features from coarse to fine at different levels, thereby effectively processing targets of various sizes. In the target detection task of deep learning, feature maps at different levels contain features of different scales and different semantic information. The low-level feature map has high resolution and contains more detailed information, which is critical for locating small targets, while the high-level feature map has low resolution but rich semantic information, which helps classify and identify targets.

The feature map levels used in the feature pyramid network in the original model are levels 3, 4, and 5. It is improved to 2, 3, and 4 layers according to the characteristics of the dataset. By introducing low-level features, the model can have a more sensitive representation of small targets. When detecting small targets, this enhanced feature information can be used to achieve feature fusion at different scales, thereby enhancing the detection ability of small targets. In addition, the step size of the feature pyramid structure determines the downsampling rate of the feature map in the pyramid structure. Adjusting the step size can change the resolution of the feature map, thereby affecting the detection ability of small targets. According to the characteristics of the HIT-UAV dataset, the step size of the feature pyramid network is adjusted from the original large step size to 4, 8, 16, and 32. A smaller step size means that the feature map has a higher resolution and can retain more detailed information. In addition, adjusting the step size can make different levels in the pyramid structure better match targets of different scales. This allows the model to detect targets at multiple scales simultaneously, especially for small targets, where the model can utilize high-resolution feature maps for more precise positioning.

#### 3.1.2. Neck Network: LCPAN

The neck network LCPAN combines the LCNet module and adopts the structure of a path aggregation network (PAN) to process image features. LCPAN receives multiple parameter configurations input from LCNet through a dictionary. The upsample in LCPAN is an upsampling layer used to increase the feature map size in the upper-lower path, wherein top_down_blocks is a layer list containing convolutional blocks in the upper-lower path for processing the upsampled feature map and the feature map from the lower path, whereby downsamples and bottom_up_blocks contain downsampling and convolutional blocks in the up-down path, respectively, for processing feature maps of different scales. The forward function defines the process of data passing through the LCPAN network, such that the data first processes the input features through conv_t, then performs feature fusion through the upper-lower path and the lower-upper path, and finally outputs a multi-scale feature map.

#### 3.1.3. Head Network: PicoHeadV2

The head part uses the PicoHeadV2 network, which is responsible for the classification and regression output of the final target. First, the features extracted from the feature pyramid structure are input by defining the picofeat class, and then classification and regression tasks are performed through multiple convolutional layers, followed by loss function calculation, anchor point generation, and other post-processing operations.

Picodet is a target detection algorithm without anchor boxes, while YOLO, RCNN, etc. are anchor box algorithms. Anchor boxes are used to match ground truth boxes for bounding box regression and classification loss calculations. It generates a direct impact on the model’s ability to detect targets of different sizes and locations. For the anchor box algorithm, the size distribution of the target in the dataset can be analyzed and the size of the anchor box can be manually adjusted to improve the recognition accuracy. The principle of the anchor-free algorithm is to generate anchors in each cell of the feature map to represent the target center position. The coordinates of the anchor point are generated through the orange and meshgrid functions, and then the offset of the cell is added. The generated anchor point coordinates are converted to floating point type and stacked into a two-dimensional tensor, where each row represents the (x, y) coordinate of an anchor point. Finally, all anchor points are combined into a 1D tensor and returned as the output of the method. The anchor point generation method of the anchor-free algorithm depends on the size of the input image, the step size of the feature pyramid, and the offset of the cell. Therefore, the accuracy of small target recognition can be improved by adjusting these parameters.

### 3.2. Datasets

**Figure 5:**
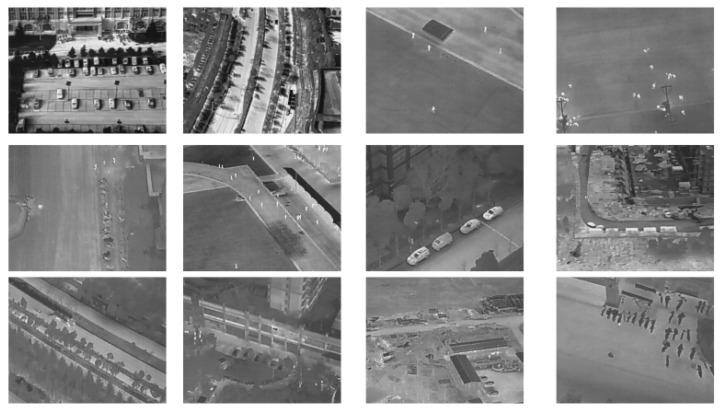
Examples of the dataset.

This paper performs model optimization and design for the HIT-UAV dataset [32]. This dataset was publicly released through Nature Research in April 2023 and is the first high-altitude UAV infrared dataset. The dataset includes images captured at different heights, viewing angles, and object types (see Figure 5 and Table 3). The dataset consists of 2898 thermal infrared images with a resolution of 640 × 512, contains 24,899 labels, and is divided into five categories, namely Person, Car, Bicycle, OtherVehicle, and don-care. Currently, only T. Aibibu et al. [33] and X. Zhao et al. [35] have launched and published model detection performance optimization work on the HIT-UAV dataset. Among them, T. Aibibu et al. [33] were the first team to optimize the recognition performance of five categories of the complete dataset, and they finally achieved an accuracy of 80%. X. Zhao et al. [35] only optimized the recognition performance of the vehicle categories in this dataset, and the accuracy was 94.5%.

**Figure 6:**
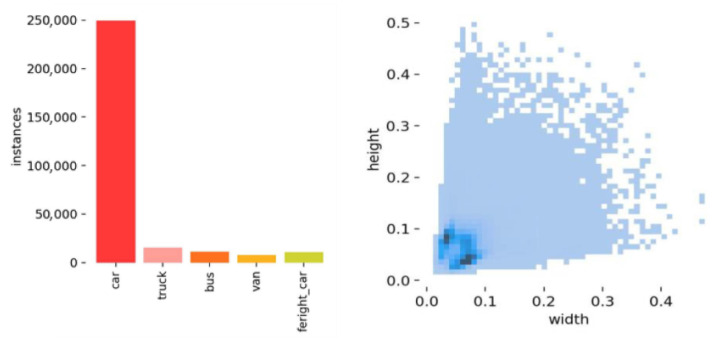
Distribution of object instances size in the HIT-UAV dataset [33].

Notably, the smallest target in the HIT-UAV dataset only accounts for 0.01% of the image pixels (see Figure 6). The dataset meets the standards for UAV detection of small infrared targets at high altitudes. In addition, we used data augmentation techniques on the basis of the HIT-UAV dataset to enrich the dataset to improve the generalization ability of the model. Specifically, it uses the methods of RandomCrop, RandomFlip, Randomdistortion, and BatchRandomResize. Among them, RandomCrop randomly cuts a part of the original image as a new training sample to increase the local feature recognition ability of the model. RandomFlip flipped the image horizontally with a probability of 0.5 to increase the symmetry recognition ability of the model. Randomdistortion is the distortion of the color balance of an image by adjusting brightness, contrast, saturation, and hue to simulate different imaging conditions. BatchRandomResize adjusts the image to a randomly selected target size within a predefined range, further increasing the diversity of the dataset. These enhancement methods are applied to the training phase to ensure that the model is robust to changes in target size, position, and imaging conditions. The NormalizeImage transform with the specified mean and standard deviation values is also applied for normalization. The combination of these data enhancement strategies can significantly improve model performance.

### 3.3. Assessment Indicators

This article uses mAP and Frames Per Second (FPS) as evaluation indicators. mAP is the common evaluation metric for measuring model detection accuracy. FPS is an evaluation indicator that measures the real-time performance of model detection. The evaluation index formula is as follows:

(3) $$\begin{array}{c} \;\mathrm{Precision}\;=\frac{\mathrm{TP}}{\mathrm{TP}+{\mathrm{FP}}^{\prime }} \end{array}$$

(4) $$\begin{array}{c} \;\mathrm{Recall}\;=\frac{\mathrm{TP}}{\mathrm{TP}+{\mathrm{FN}}^{\prime }} \end{array}$$

(5) $$\begin{array}{c} \;\text{F1-score}\;=\frac{2*P*R}{P+R}=\frac{2\mathrm{TP}}{2\mathrm{TP}+\mathrm{FP}+{\mathrm{FN}}^{\prime }} \end{array}$$

(6) $$\begin{array}{c} \mathrm{AP}=\int_{0}^{1}P\left(r\right)\mathrm{dr}, \end{array}$$

(7) $$\begin{array}{c} \mathrm{mAP}=\frac{\sum_{i=1}^{N}{\mathrm{AP}}_{i}}{N}. \end{array}$$

The terms true positive (TP), false positive (FP), and false negative (FN) are usually used to refer to correctly identified, incorrectly identified, and missed samples, respectively. N is a variable indicating the number of categories for classification.

## 4. Verifications

### 4.1. Experimental Platform and Parameter Settings

To evaluate the effectiveness of the improved model, comparison experiments and ablation experiments were designed. The hardware platform settings used during the experiment are shown in Table 4.

### 4.2. Algorithms’ Comparisons

To evaluate the usability and effectiveness of the improved model in detecting small infrared targets in UAV scenarios, comparative studies were conducted with various advanced algorithms under the same conditions. All experimental models are not trained with pre-training weights and training is restarted. The input image size of the model was adjusted to 416 × 416, the batch size was set to 16, and the number of iterations was set to 300. The training results show that compared with the original model, the improved model’s mAP increased by 7% and the frame rate increased by 31 fps. It shows that the improved model has lower model complexity and higher accuracy in UAV infrared detection of small targets. Experimental results verify the effectiveness of this method. Detailed experimental data are shown in Table 5.

**Figure 7:**
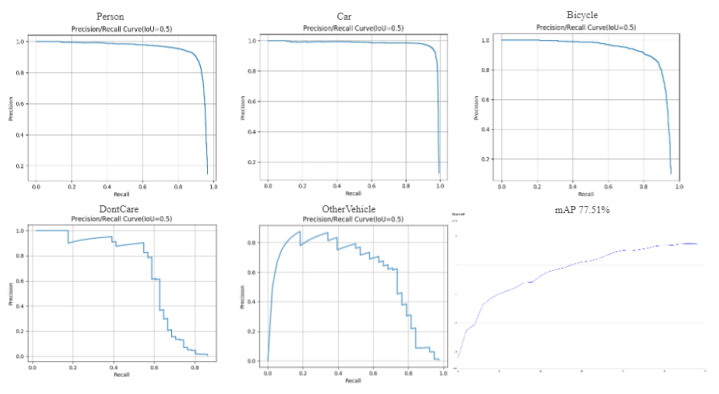
Precision-Recall Curve graphs for five categories of objects.

Figure 7 shows the Precision-Recall Curve graphs corresponding to our model’s evaluation of the five categories in the HIT-UAV dataset.

**Figure 8:**
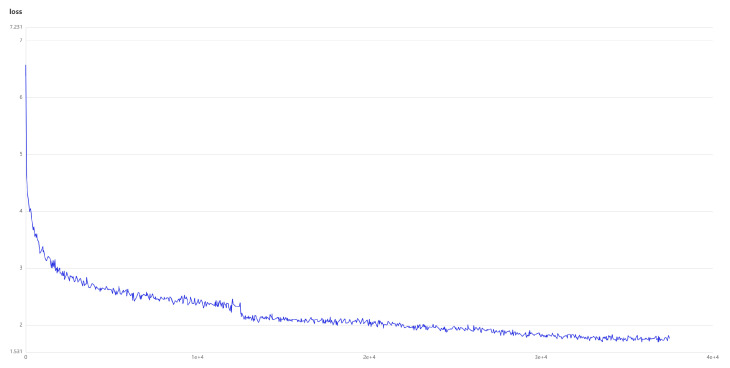
Loss function of the models.

In order to prove that the choice of the number of training cycles is correct, we give the loss function diagram of the model. As shown in Figure 8.

To further verify the reliability of the improved model, we chose to conduct comparative experiments with the YOLO series and Mobilenet models on the HIT-UAV infrared target dataset. The target height of this dataset is 60–130 m, which results in complex and changeable image backgrounds and large changes in the target scale. Furthermore, most targets are small, which poses a great challenge to detection. The results in Table 6 show that the improved model has significant advantages in detecting small infrared targets for UAV. Specifically, when the input resolution is 416 × 416, compared with Mobilenet-v1, the improved model improves mAP by 43% and improves frame rate by 15 fps. Compared with YOLOv4-tiny, the improved model increased mAP by 26%. Compared with Faster RCNN, the improved model improved mAP by 31%. The above experimental results show that the improved model proposed in this article can achieve high detection accuracy and accuracy when identifying small infrared targets, even in complex ground backgrounds, regardless of the angle and height of the drone collection.

**Figure 9:**
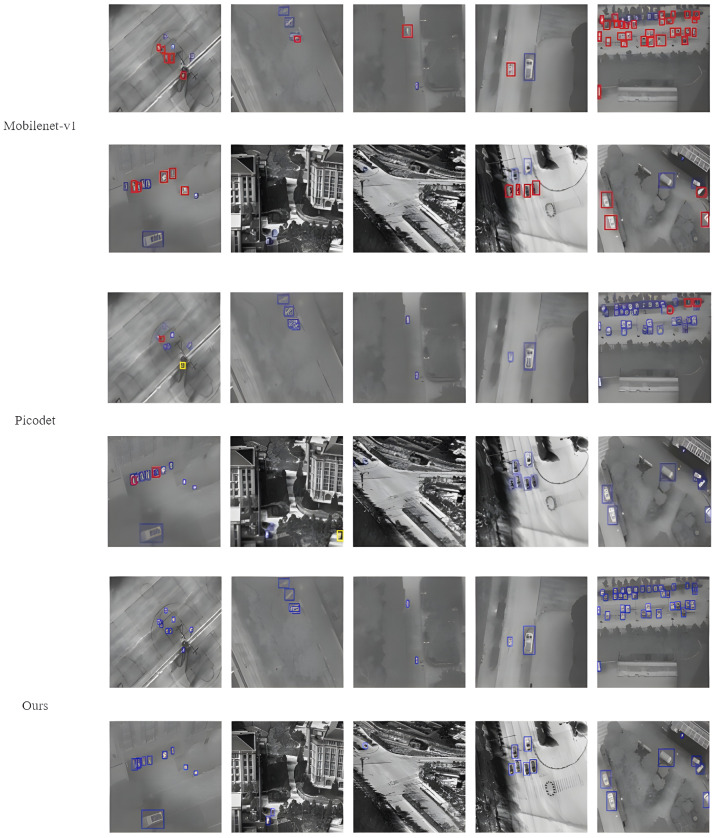
The outcomes of the various algorithms are presented through visualization.

The results of different algorithms are shown in Figure 9, wherein the improved algorithm has achieved significant improvements in UAV infrared small target detection. Our method improves detection accuracy and reduces the number of missed detections by learning the characteristic information of small infrared targets of different scales.

In Figure 9, true positive (TP) instances are represented by a blue box. False positive (FP) and false negative (FN) instances are represented by yellow and red boxes, respectively. Both Mobilenet-v1 algorithm and unimproved Picodet algorithm have false detection and missing detection. Compared with the two algorithms, our algorithm can solve these error detection cases well.

### 4.3. Ablation Studies and Analysis

To verify the effectiveness of the improved model proposed in this article, a series of ablation experiments were conducted on the HIT-UAV dataset to evaluate the impact of each module on the improved model. Specifically, the input image size used in the ablation experiment was 416 × 416, the batch size was 16, and each network was trained for 300 epochs. The experimental results are shown in Table 7.

As can be seen from Table 7, under the same settings, the detection accuracy of the model after optimizing the network structure is significantly improved compared to the original model. Adding the SE module is beneficial to the increase of mAP, and does not cause the real-time frame rate to decrease. Improving the feature pyramid network structure increases mAP by 1.55%. Improving the weight of the loss function can also significantly improve mAP. In addition, better model performance is obtained through cosine decay linear warm-up learning strategies and optimizer improvements. Comparing the performance of the improved algorithm with the original algorithm, the real-time detection frame rate increased by 31 fps, and the mAP increased by 7%, which greatly improved the efficiency of target detection.

## 5. Conclusions

This paper proposes an improved algorithm for small-scale and multi-scale infrared small-target detection in UAV scenes. In this method, the LCNet-based backbone network and the Picodet model are used as the basic framework. The design of the network reduces the complexity of the model and enhances the fusion of feature information, making it more compatible with mobile devices. In addition, by improving the structure of the SE module in the lightweight LCPAN network and the feature pyramid structure, the generalization ability of the network is improved, and the feature extraction and fusion of small infrared targets are improved. Given the particularity of small targets in UAV aerial images, the learning strategy of cosine attenuation and linear preheating is adopted to make the model more suitable for infrared small target detection in UAV scenes. Experimental results show that compared with the original model, the method proposed in this article has great improvement in real-time performance, and the frame rate is increased by 31 fps. A high frame rate means that the camera is able to capture more image frames per second, increasing the possibility of capturing dynamic changes in the target. Our work increased the frame rate by 31 fps, allowing for more accurate capture of fast-moving objects, such as fast-moving vehicles or pedestrians. Higher frame rate can reduce motion blur and improve response time to fast events, which increases detection accuracy and reliability. In addition, the average precision (mAP) is improved by 7%. The higher the mAP is, the higher the average accuracy of the algorithm in detecting various targets, and the fewer the cases of false detection and missed detection. The 7% increase in mAP means that our algorithm has better accuracy and reliability in detecting small infrared targets, such that the improved model is more real-time and accurate in UAV infrared small target detection and has practical application value.

However, it should be noted that although the network we designed reduces the model complexity and improves the real-time performance, it also sacrifices the detection accuracy to some extent. In some application scenarios where accuracy is very high, more complex models may be required to obtain higher detection accuracy. The method in this paper is mainly optimized for the UAV infrared small target detection scenario. Therefore, when applied to other types of object detection tasks or images of different scenes, further adjustments and optimizations may be required. Our approach achieved significant performance improvements on the HIT-UAV dataset, but may not perform well on other datasets, as the heterogeneity and complexity of the dataset may affect the model’s generalization ability.

In this paper, the algorithm is deployed on a CPU+GPU platform. In practical applications, it is often necessary to deploy on the UAV mobile platform, such as FPGA, SOC, ARM, and other hardware platforms. However, the same model on different hardware platforms may have a 1000-fold difference in inference performance. In order to apply the proposed algorithm to the actual scenarios of UAVS, further research directions include: (1) further lightweight model, (2) hardware adaptation design, (3) implementation of hardware parallel acceleration algorithm, etc. For the lightweight model method, we can use model pruning and quantization technology to reduce the computational complexity of the model by removing the redundant part of the model, so as to further reduce the size of the model and improve the running speed. Hardware adaptation design is designed for hardware deployment because some hardware platforms, such as FPGA and SOC, may not support the operators used in the model implementation. The hardware parallel acceleration algorithm is realized by using some parallel acceleration algorithms in the hardware platform.

## Figures and Tables

**Table 1:** Network structure parameters before modification.

Operator	Kernel Size	Stride	SE Model
Conv2D	3 × 3	2	-
DepthSepConv	3 × 3	1	-
DepthSepConv	3 × 3	2	-
DepthSepConv	3 × 3	1	-
DepthSepConv	3 × 3	2	-
DepthSepConv	3 × 3	1	-
DepthSepConv	3 × 3	2	-
5 × DepthSepConv	5 × 5	1	-
DepthSepConv	5 × 5	2	✓
DepthSepConv	5 × 5	1	✓
GAP	7 × 7	1	-
Conv2D, NBN	1 × 1	1	-

**Table 2:** Improved network structure parameters.

Operator	Kernel Size	Stride	SE Model
Conv2D	3 × 3	2	-
DepthSepConv	3 × 3	1	-
DepthSepConv	3 × 3	2	-
DepthSepConv	3 × 3	1	-
DepthSepConv	3 × 3	2	-
DepthSepConv	3 × 3	1	-
DepthSepConv	3 × 3	2	-
3 × DepthSepConv	5 × 5	1	-
2 × DepthSepConv	5 × 5	1	✓
DepthSepConv	5 × 5	2	✓
DepthSepConv	5 × 5	1	✓
GAP	7 × 7	1	-
Conv2D, NBN	1 × 1	1	-

**Table 3:** Number of tags for small, medium, and large targets in HIT-UAV dataset [35].

	Small (0,32×32)	Medium (32×32,96×96)	Large (96×96,640×512)
HIT-UAV	17,118	7249	268
Train set	12,045	5205	268
Test set	3331	1379	70
Validation set	1742	665	46

**Table 4:** Experimental platform information.

Names	Related Configurations
Graphics processing unit	NVIDIA RTX4060
Central processing unit	AMD7945
GPU memory size	16G
Operating system	Win11
Computing platform	CUDA11.2
Deep learning framework	paddlepaddle

**Table 5:** Performance comparison table between Picodets and Ours.

	mAP (%)	FPS
Picodet-s	70.57	65.76
Ours	77.51	96.78

**Table 6:** Experimental results comparison with others.

Model	Person F1	Car F1	Bicycle F1	Other Vehicle F1	Dont Care F1	mAP (%)	FPS
Mobilenet-v1	0.291	0.692	0.329	0.571	0.107	34.15	81.13
Picodet	0.747	0.924	0.754	0.694	0.674	70.57	65.76
YOLOv4-tiny [32]	-	-	-	-	-	50.38	-
Faster RCNN [33]	-	-	-	-	-	45.50	-
Ours	0.904	0.953	0.868	0.675	0.683	77.51	96.78

**Table 7:** Ablation experiment results.

Picodet	SE Model	Improved Feature Pyramid Structure	Improve Learning Strategies and Optimizers	Improve Loss Function Weights	mAP (%)	FPS
✓	-	-	-	-	57.96	128.58
✓	-	-	✓	-	70.57	65.76
✓	-	✓	✓	-	72.12	77.02
✓	Block4 + Block5 (all)	✓	✓	-	72.68	78.80
✓	Block5 × 1	✓	✓	✓	74.68	100.93
✓	Block5 × 2	✓	✓	✓	77.51	96.78

## Data Availability

No new data were created.
